# Mechanism of Action and Translational Potential of (*S*)-Meclizine in Preemptive Prophylaxis Against Stroke

**DOI:** 10.1161/STROKEAHA.123.044397

**Published:** 2024-04-04

**Authors:** Jeong Hyun Lee, Vishal M. Gohil, Pedram Heidari, Jessica L. Seidel, Mohammad Zulkifli, Ying Wei, Yuhua Ji, Ali Daneshmand, Umar Mahmood, Clary B. Clish, Vamsi K. Mootha, Cenk Ayata

**Affiliations:** Neurovascular Research Unit, Department of Radiology, Massachusetts General Hospital, Harvard Medical School, Charlestown (J.H.L., P.H., J.L.S., Y.W., A.D., U.M., C.A.).; Therapeutics and Biotechnology Division, Korea Research Institute of Chemical Technology, Graduate School of New Drug Discovery and Development, Chungnam National University, Daejeon, South Korea (J.H.L.).; Department of Biochemistry and Biophysics, Texas A&M University, College Station (V.M.G., M.Z.).; Grace Science, LLC, Menlo Park, CA (Y.J.).; Broad Institute of MIT and Harvard, Cambridge, MA (C.B.C., V.K.M.).; Howard Hughes Medical Institute and Department of Molecular Biology, Massachusetts General Hospital, Boston (V.K.M.).; Department of Systems Biology, Harvard Medical School, Boston, MA (V.K.M.).

**Keywords:** chemical preconditioning, ischemic injury, middle cerebral artery occlusion, neuroprotection, preemptive prophylaxis

## Abstract

**BACKGROUND:**

Mild chemical inhibition of mitochondrial respiration can confer resilience against a subsequent stroke or myocardial infarction, also known as preconditioning. However, the lack of chemicals that can safely inhibit mitochondrial respiration has impeded the clinical translation of the preconditioning concept. We previously showed that meclizine, an over-the-counter antivertigo drug, can toggle metabolism from mitochondrial respiration toward glycolysis and protect against ischemia-reperfusion injury in the brain, heart, and kidney. Here, we examine the mechanism of action of meclizine and report the efficacy and improved safety of the *(S*) enantiomer.

**METHODS:**

We determined the anoxic depolarization latency, tissue and neurological outcomes, and glucose uptake using micro–positron emission tomography after transient middle cerebral artery occlusion in mice pretreated (−17 and −3 hours) with either vehicle or meclizine. To exclude a direct effect on tissue excitability, we also examined spreading depression susceptibility. Furthermore, we accomplished the chiral synthesis of *(R*)- and *(S*)-meclizine and compared their effects on oxygen consumption and histamine H1 receptor binding along with their brain concentrations.

**RESULTS:**

Micro–positron emission tomography showed meclizine increases glucose uptake in the ischemic penumbra, providing the first in vivo evidence that the neuroprotective effect of meclizine indeed stems from its ability to toggle metabolism toward glycolysis. Consistent with reduced reliance on oxidative phosphorylation to sustain the metabolism, meclizine delayed anoxic depolarization onset after middle cerebral artery occlusion. Moreover, the *(S*) enantiomer showed reduced H1 receptor binding, a dose-limiting side effect for the racemate, but retained its effect on mitochondrial respiration. *(S*)-meclizine was at least as efficacious as the racemate in delaying anoxic depolarization onset and decreasing infarct volumes after middle cerebral artery occlusion.

**CONCLUSIONS:**

Our data identify *(S*)-meclizine as a promising new drug candidate with high translational potential as a chemical preconditioning agent for preemptive prophylaxis in patients with high imminent stroke or myocardial infarction risk.

It has long been appreciated that mild inhibition of mitochondrial respiration hours to days before an ischemic insult induces a protective state known as ischemic tolerance or preconditioning.^1–3^ The heavy reliance of the brain on oxidative phosphorylation to sustain its energy needs at rest renders it highly susceptible to injury under hypoxic or ischemic conditions such as cardiac arrest or focal arterial occlusions. Early studies demonstrated that short, sublethal bouts of ischemia can confer protection against longer, more sustained vessel occlusion, a concept termed ischemic preconditioning. Subsequently, it was appreciated that mild, chemical inhibition of the mitochondrial oxidative phosphorylation system, leading to a shift in metabolism toward glycolysis, could enhance cell survival during subsequent hypoxic-ischemic insults.^1–3^ This form of chemical preconditioning can in principle be invaluable as preemptive prophylaxis against spatiotemporally predictable hypoxic-ischemic events, such as cardiovascular or cerebrovascular interventions that carry high stroke risk. If clinically well tolerated, chemical preconditioning could also be implemented as long-term stroke prophylaxis.

Although known mitochondrial respiratory chain inhibitors were shown to induce ischemic tolerance in the laboratory, their narrow therapeutic index and potentially harmful side effects have precluded clinical translation. We previously designed a nutrient-sensitized screen for novel modulators of mitochondrial respiration with favorable toxicity profiles and discovered that meclizine, the over-the-counter antivertigo drug, shifts cell metabolism from oxidative phosphorylation toward glycolysis independent of its antimuscarinic and antihistaminic effects.^4^ Consistent with its ability to attenuate the mitochondrial respiration,^5^ meclizine protected the brain, heart, and kidney against the ischemic injury.^4,6^ However, in vivo evidence directly linking its metabolic effect to neuroprotection has been missing. Moreover, the antimuscarinic and antihistaminic side effects of meclizine at doses required to achieve chemical preconditioning and neuroprotection have thus far precluded clinical development for this indication.

Here, we provide the first direct in vivo evidence that the neuroprotective effect of meclizine indeed stems from its ability to toggle metabolism. Meclizine is a racemic mixture of equal amounts of (*S*)-meclizine and (*R*)-meclizine. We synthesized these 2 enantiomers and showed that while both (*R*)- and (*S*)-meclizine are equipotent in inhibiting mitochondrial respiration and inducing ischemic tolerance, the (*S*)-enantiomer has a better safety profile because of substantially weaker antihistaminic activity, making it an exciting candidate for cerebroprotection.

## METHODS

### Data Availability

The data supporting the findings of this study are available from the corresponding author upon reasonable request.

### Experimental Animals

Experiments were approved by the MGH Institutional Animal Care and Use Committee and carried out per the Guide for Care and Use of Laboratory Animals (National Institutes of Health Publication No. 85 to 23, 1996). The design and report of the study were in accordance with the Animal Research: Reporting of In Vivo Experiments guidelines.^7^ Mice (2 to 3 months old, C57BL/6J, only males to avoid the confounding effects of the estrus cycle, Charles River Laboratories), housed on a 12-hour light/dark cycle and fed ad libitum.

### Middle Cerebral Artery Occlusion

The experimenter was blinded to treatment groups. Mice were anesthetized with isoflurane (2.5% induction, 1.5% maintenance, in 70% N_2_O/30% O_2_) and subjected to 1-hour transient middle cerebral artery occlusion (MCAO) using an intraluminal filament (7021PK, Doccol Corporation, Sharon, MA) inserted through the external carotid artery. Regional cerebral blood flow (CBF) was monitored using a laser Doppler probe placed over the core middle cerebral artery territory. Rectal temperature was controlled at 37 °C by a servocontrolled heating pad. One day after MCAO, animals were sacrificed under deep isoflurane anesthesia, brains removed, and 1-mm-thick coronal slices were incubated in 2% triphenyl tetrazolium chloride in PBS (Sigma-Aldrich) at 37 °C for 10 minutes. Slices were imaged, and the areas of the infarct, as well as the ipsilateral and contralateral hemispheres in each slice, were integrated to obtain volumes (ImageJ, National Institutes of Health, Bethesda, MD). Infarct volume was corrected for ischemic swelling using (contralateral hemisphere−[ipsilateral hemisphere−infarct]). There was no premature mortality in any group. Neurological outcomes were scored as a secondary end point 24 hours after reperfusion, using a 6-point scale: 0, normal; 1, forepaw monoparesis; 2, circling to left; 3, falling to left; 4, no spontaneous walking and depressed consciousness. This scoring system, although widely used, shows high variability and low sensitivity and requires nonparametric testing. Therefore, the study was not powered to achieve statistical significance in this metric.

### Positron Emission Tomography

After MCAO, mice were positioned in the scanner to have both the heart and the brain in the field of view. The 2-deoxy-2-[fluorine-18]fluoro-D-glucose (^18^F-FDG, 385±31 μCi in 150 to 200 μL final volume; Sofie Pharmaceuticals, Haverhill, MA) was injected via the lateral tail vein. Dynamic positron emission tomography (PET) data were acquired in list mode for 60 minutes beginning immediately before injection of ^18^F-FDG (Argus micro-PET; Sedecal, Madrid, Spain). The data were then reframed in 40 15-second, 20 30-second, 16 60-second, and 16 90-second frames. The final 6 minutes of the acquisition were reframed separately to produce a high-count static image. Scans were reconstructed using 3-dimensional ordered subset expectation maximization (3D-OSEM) reconstruction in 2 iterations and 16 subsets and corrected for randoms and scatter. The reconstructed images were loaded in Amid and 3-dimensional regions of interest (ROI) were manually drawn in the descending aorta, infarct core, penumbra, and contralateral cerebral cortex. Mean-standardized ^18^F-FDG uptake values (SUV_mean_) in the ROIs were calculated. The input function was measured from the aortic ROI. Time-activity curves were plotted for the core, penumbra, and normal cortex.

Tracer injection quality can affect the dynamics of tracer uptake in the tissue; tracer extravasation in particular leads to blunted peak blood pool time-activity curves and prolonged presence of tracer in the blood pool (ie, input function). This alters the pharmacokinetics of the tracer and confounds the tissue uptake profile. To assess the quality of the tracer injection, the plasma uptake values at each frame were normalized to peak plasma uptake in each mouse. With good injection quality, normalized plasma uptake is expected to remain below 45% of the peak uptake after reaching the steady state at 25 minutes. Therefore, mice that consistently had a normalized plasma uptake above 45% were excluded (2 out of 11 in the meclizine group and 0 out of 8 mice in the control group).

Given that SUV_mean_ is a function of tissue perfusion rate and tracer uptake rate, we sought to analyze the data using a model that excludes the effect of tissue perfusion. Since ^18^F-FDG uptake follows an irreversible 2-compartment tissue model,^8^ we employed a Patlak graphical plot and calculated the net tracer influx rate (*Ki*, mL plasma/mL tissue/min), which represents the tissue tracer uptake rate independent of perfusion and reflects the rate of glucose utilization by the tissue.^9,10^ Since steady state was achieved in <25 minutes, we used a 25-minute cutoff to fit the *Ki*.

### Spreading Depolarization Susceptibility Testing

As described previously,^11^ 3 burr holes were drilled under saline cooling at the following coordinates (mm from bregma): 3.5 posterior, 2 lateral (2 mm diameter for electric stimulation and KCl application onto occipital cortex); 1.5 posterior, 2 lateral (1 mm diameter, recording site 1); 0.5 anterior, 2 lateral (1 mm diameter, recording site 2). The dura was kept intact to minimize trauma. Two glass capillary microelectrodes were placed to record extracellular steady (DC) potential and electrocorticogram. The electric spreading depolarization (SD) threshold was determined by escalating intensity cathodal square pulses (10–8000 μC) via a bipolar electrode placed on the occipital cortex and then a 1-mm cotton ball soaked in 300 mmol/L KCl was topically applied for 1 hour to record the frequency of evoked SDs. SD frequency and threshold were taken as primary end points. The amplitude, propagation speed (distance/latency between the 2 recording electrodes), duration at half-amplitude of the first SD, and the cumulative durations of all SDs triggered by topical KCl were also measured as secondary end points.

#### Chiral Synthesis of (R)- and (S)-Meclizine

Chiral synthesis of meclizine using enantiomers of tartaric acid was achieved in 4 steps:

The synthesis of (*R*)- and (*S*)-meclizine was achieved in 4 steps, starting with chiral resolution of racemic 4-chlorobenzhydrylamine using D- and L-enantiomers of tartaric acid.

#### Step 1

(*R*) and (*S*)-4-chlorobenzhydrylamine

The racemic 4-chlorobenzhydrylamine was treated with D-tartaric acid and then sequentially recrystallized 10 times from water as described previously^12^ to provide (*S*)- 4-chlorobenzhydrylamine of high optical purity (ee>98%, according to chiral HPLC). In a similar manner, treatment of racemic 4-chlorobenzhydrylamine with L-tartaric acid provided (*R*)- 4-chlorobenzhydrylamine of high optical purity (>98%, according to chiral HPLC).

#### Step 2

(*R*) and (*S*)1-((4-chlorophenyl; phenyl)methyl) N-tosylpiperazine was synthesized by reacting each enantiomer of 4-chlorobenzhydrylamine (100 mg, 0.45 mmol) with *N,N*-bis(2-chloroethyl)-4-methylbenzenesulfonamide (150 mg, 0.50 mmol) in the presence of a base.

#### Step 3

(*R*) and (*S*)1-((4-chlorophenyl; phenyl)methyl)piperazine was prepared as described previously^13^ by deprotecting *N*-tosyl group in the presence of *p*-nitrobenzoic acid.

#### Step 4

(*R*)-meclizine and (*S*)-meclizine

A mixture containing (*S*)-1-[(-4-chlorophenyl)phenylmethyl]piperazine (50 mg, 0.18 mmol), 3-methylphenylmethyl bromide (0.025 mL, 0.19 mmol), potassium carbonate (80 mg, 0.56 mmol) and methanol (3 mL) was stirred under reflux for overnight. The mixture was then cooled to room temperature and filtered. The filtrate was concentrated and then purified by chromatography over silica-gel to afford 10 mg of (*S*)-meclizine as a yellow oil, which was further converted to its hydrochloride salt. MS: 391 (M+1); ee: >99% (retention time=22.6 minutes; CHIRALPAK OD-H column; mobile phase: Hexanes 0.1% diethyl amine [DEA]).

(*R*)-meclizine was synthesized in the same manner by using (*R*)-1-[(-4-chlorophenyl)phenylmethyl]piperazine.

### In Vitro Assays

Oxygen consumption rate and extracellular acidification rate measurements were performed as described previously.^4^ Briefly, HEK 293MSR cells, obtained from Prof. Stephen Haggarty, Massachusetts General Hospital, Boston, were seeded in XF24 cell culture microplates at 50 000 cells/well in DMEM high glucose media, supplemented with 10% fetal bovine serum. The cells were incubated overnight at 37 °C with 5% CO_2_. Before the measurements, the growth medium was replaced with ≈925 μL of assay medium devoid of serum, and cells were incubated at 37 °C for 30 minutes in the assay medium before measurements. The measurements were made every 6 minutes after a 2-minute mix and 2-minute wait period. Three baseline measurements were recorded before the addition of (*R*)- or (*S*)-meclizine. Stock solutions were made in 100% DMSO, which was diluted to a specified concentration in the assay medium, and pH was adjusted to 7.4 by 1 N NaOH.

To measure cell viability, HEK 293MSR cells were seeded at a density of 20 000 cells/well in a 96-well plate in DMEM high glucose media, supplemented with 10% fetal bovine serum, and incubated overnight at 37 °C with 5% CO_2_. Spent media from each well were replaced using growth media containing indicated concentrations of drugs, and the plates were incubated for another 6 hours. Cell viability was then assessed using the CellTiter-Glo cell viability assay (Promega). Cell viability of murine ST*Hdh*^Q111/111^ striatal cells was also measured by CellTiter-Glo cell viability assay (Promega). Briefly, cells were cultured in serum-containing media for 24 hours in 96-well plates at 20 000 cells/well, followed by 24 hours of drug treatment in serum-free media before performing the viability assay.

In vitro binding of *R*- and *(S*)-meclizine to human H1 receptors was determined by competitive binding assays in the presence of 3 nmol/L [^3^H]pyrilamine (CEREP Laboratories, France). Nonspecific binding was determined in the presence of excess (1 μM) unlabeled pyrilamine. IC_50_ was determined by nonlinear regression analysis of the competition curves.

Brain meclizine and (*S*)-meclizine levels were measured after daily intraperitoneal injections for 5 days. Samples were prepared by adding a 40× extraction buffer (75% acetonitrile, 25% methanol, 0.2% formic acid) containing 10% water to pulverized brain tissue (40 μL/mg of tissue). The mixture was homogenized for 30 seconds followed by 30 seconds of vortexing. The sample was centrifuged at 10 000g for 10 minutes at 4 °C, and the supernatant was analyzed by using hydrophobic interaction liquid chromatography followed by mass spectrometry. The MS/MS spectrum shows a dominant product ion at m/z 201, and we selected the 391/201 multiple reaction monitoring (MRM) transition for quantitation of meclizine isomers, similar to LC-MS methods developed by others.^14,15^

### Data Analyses and Statistics

Mice were randomly picked from the cages and allocated to treatment arms; no specific randomization tool was used. All procedures and data analyses were performed blinded. The only a priori exclusion criterion was technical failures. Sample sizes are indicated in the Figures. Initial in vivo sample sizes were selected empirically to achieve at least 80% power to detect a 30% effect size, assuming an SD of 25% of the mean (α=0.05) and adjusted in subsequent experiments to reflect the initial experience for each readout. Statistical comparisons were designed, a priori, to compare vehicle versus treatment arms. Normal distribution was tested by the Shapiro-Wilk test. All statistical tests are indicated in Figure legends (Prism 9, GraphPad Software Inc, CA). Data are shown as whisker-box plots (whiskers, full range; box, interquartile range; +, mean; horizontal line, median) or mean±SEM.

## RESULTS

### Meclizine Increases Glucose Utilization in the Ischemic Penumbra

**Figure 1.**
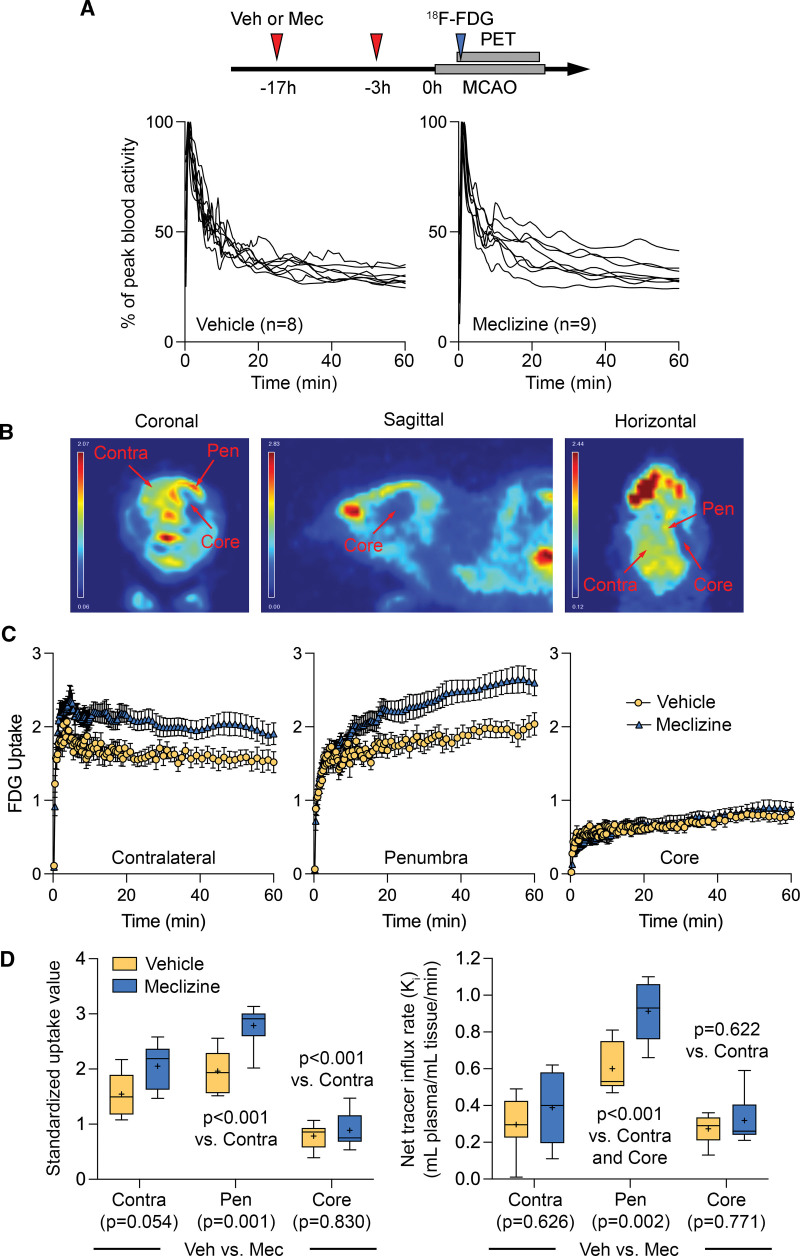
**Effect of meclizine on ^18^F-FDG PET (2-deoxy-2-[fluorine-18]fluoro-D-glucose positron emission tomography) glucose uptake during acute middle cerebral artery occlusion (MCAO). A**, Representative ^18^F-FDG PET images during middle cerebral artery occlusion show increased glucose uptake in the ischemic penumbra (Pen) and decreased uptake in the ischemic core compared with the contralateral nonischemic brain. The typically very high ocular glucose uptake and its relative reduction ipsilateral to the middle cerebral artery occlusion are conspicuously visible, the latter presumably due to carotid artery occlusion performed as part of the procedure. **B**, The time course of blood activity in the vehicle and meclizine arms. **C**, The time-activity curves show regional differences as well as the effect of meclizine. Two-way ANOVA. **D**, Standardized ^18^F-FDG uptake and the net tracer influx rate are shown for the 3 regions of interest and treatment arms. *P* values represent post hoc comparisons among the regions (shown on the graph) and between treatment arms in each region (shown at the bottom of each region). Two-way ANOVA followed by Šídák multiple comparisons.

We first sought to obtain direct evidence of a metabolic shift using ^18^F-FDG PET after pretreatment (17 and 3 hours before MCAO) with meclizine (Figure 1A). The blood time-activity curves were within the anticipated range and similar between the treatment arms (n=8 vehicle, 9 meclizine; Figure 1A). Glucose utilization showed major regional heterogeneity during acute MCAO among the ischemic core and penumbra and the contralateral brain (Figure 1B). The time-activity curves reflected these regional and treatment-related differences in tissue uptake (Figure 1C).

The SUV_mean_ was significantly reduced in the ischemic core and elevated in the ischemic penumbra compared with the contralateral nonischemic brain in both control and meclizine arms (*P*<0.001 versus contralateral ROI, 2-way ANOVA followed by Tukey pairwise multiple comparisons, independent variables ROI and treatment arm; Figure 1D, left). Since SUV_mean_ is a function of both tissue perfusion and glucose utilization, we used compartmental modeling to calculate the rate of glucose utilization independent of tissue perfusion (ie, Ki). When corrected for tissue perfusion, the *Ki* was significantly elevated in the ischemic penumbra compared with both the ischemic core and the contralateral nonischemic brain in both control and meclizine arms (*P*<0.001 versus ischemic core and contralateral brain; Figure 1D, right); the *Ki* in the core and the nonischemic brain did not significantly differ (*P*=0.622).

Pretreatment with meclizine elevated the SUV_mean_ in the penumbra by 42% compared with controls (*P*=0.001), and strongly tended to increase it in the contralateral brain (*P*=0.054), but not in the ischemic core (*P*=0.830; Figure 1D, left). When corrected for tissue perfusion, the *Ki* in the penumbra was 52% higher in the meclizine group compared with controls (*P*=0.002) but did not differ between the treatment arms in the contralateral brain (*P*=0.626) or the infarct core (*P*=0.771; Figure 1D, right). These data provide the first in vivo evidence for the metabolic shift towards anaerobic glycolysis induced by meclizine and showed that this effect is most prominent in the ischemic penumbra, explaining its neuroprotective efficacy.

### Meclizine Delays Anoxic Depolarization After Ischemia Onset

**Figure 2.**
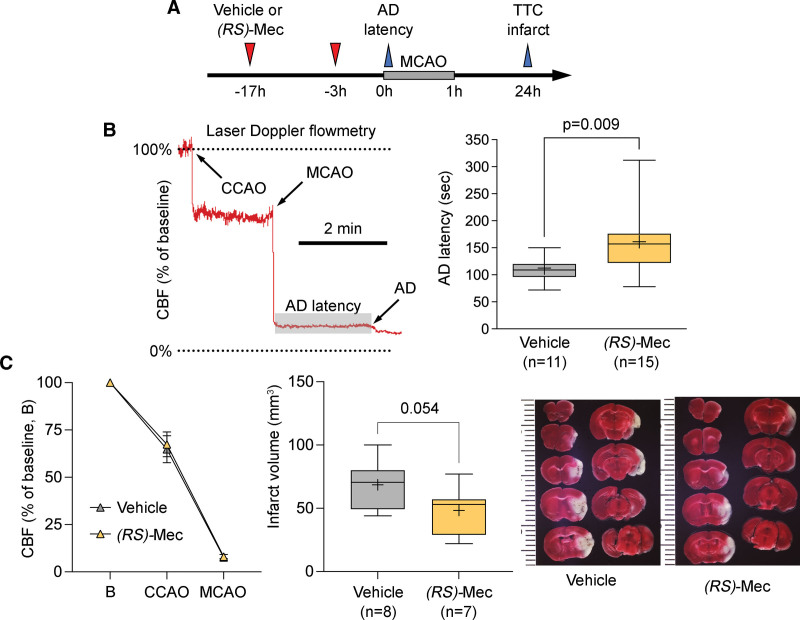
**Effects of meclizine on anoxic depolarization latency, cerebral blood flow (CBF), and infarct volume in the filament middle cerebral artery occlusion (MCAO) model. A**, Experimental timeline shows the treatment protocol and the timing of data collection for anoxic depolarization (AD) latency and infarct volume using triphenyl tetrazolium chloride (TTC) staining. **B**, A representative CBF tracing shows the common carotid artery occlusion (CCAO) and MCAO time, and the stepwise CBF reduction at the onset of AD. The shaded area shows the measurement of AD latency. The right shows the prolonged AD latency in the meclizine arm (unpaired *t* test). **C**, The left shows nearly identical CBF reductions upon CCAO and MCAO between meclizine and vehicle arms. The right shows smaller infarcts in the meclizine arm (unpaired *t* test).

We next tested whether the metabolic shift from oxidative phosphorylation to glycolysis renders the tissue more resilient to ischemia by measuring the time it takes for brain tissue to lose membrane potentials and undergo anoxic depolarization (AD) upon MCAO, reflecting depressed Na^+^/K^+^-ATPase activity due to declining tissue ATP. After pretreatment with vehicle or meclizine (100 mg/kg; Figure 2A), we induced MCAO under laser Doppler CBF monitoring in the severely ischemic core and used the characteristic CBF surrogate of AD, a sudden stepwise reduction in CBF, to mark the onset of AD (Figure 2B, left). Pretreatment with meclizine significantly delayed AD onset compared with vehicle (*P*=0.009; Figure 2B, right). Although the CBF reduction after common carotid artery occlusion or MCAO did not differ between the treatment arms (Figure 2C, left panel), infarct volumes, corrected for ischemic swelling (ie, indirect infarct), strongly tended to be smaller in the meclizine arm (*P*=0.054). Neurological deficit scores did not differ between the treatment arms (2.00 [1.00 to 2.75] versus 2.00 [1.00 to 3.00] vehicle and meclizine, respectively; *P*=0.857, Mann-Whitney *U* test).

**Figure 3.**
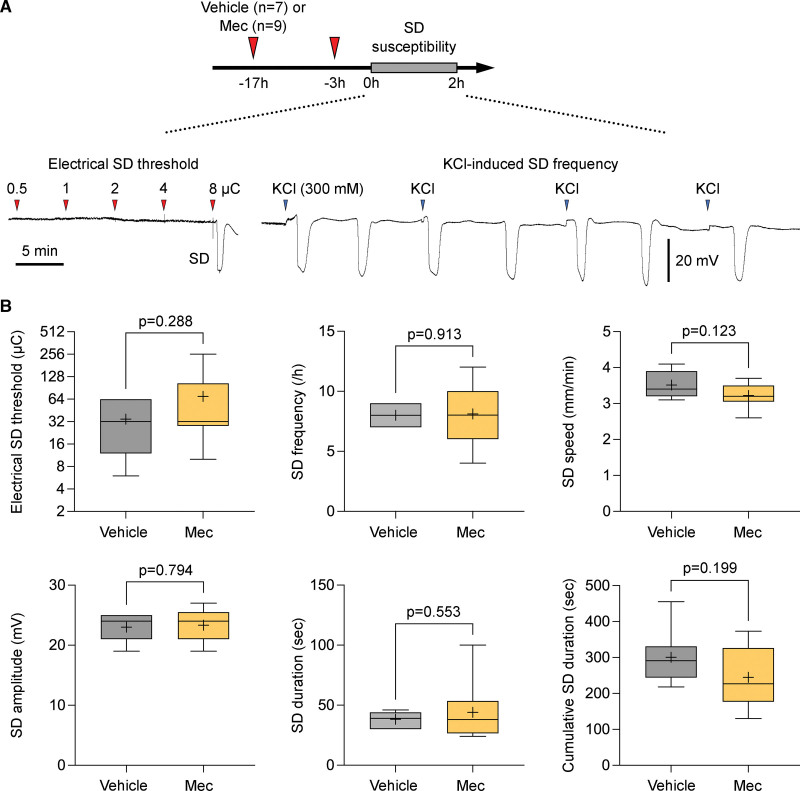
**Effects of meclizine on spreading depolarization susceptibility. A**, Representative tracings of extracellular slow (ie, direct-coupled [DC]) potential show spreading depolarization (SD) susceptibility assessment, including electrical stimulation intensity threshold (microcoulomb, μC) for SD induction (**left**) and the frequency of topical KCl application-induced SDs. Each negative deflection on the DC tracing represents an SD. **B**, The effect of meclizine on SD susceptibility attributes, including the threshold, frequency, speed, amplitude, and duration of a single SD as well as cumulative duration of all triggered SDs (unpaired *t* test).

To rule out a direct effect on tissue excitability, in a separate cohort of mice we tested meclizine on the susceptibility of nonischemic brain tissue to SD and did not find any effect (Figure 3). Pretreatment with meclizine (n=9) did not significantly affect the electrical stimulation intensity required to trigger an SD or the frequency of SDs triggered by continuous topical application of concentrated KCl solution (300 mmol/L) compared with vehicle (n=7). The SD propagation speed, amplitude, and duration were also not altered. Similar data were obtained in the rat (n=14 vehicle, 8 meclizine; data not shown). Altogether these data strongly supported metabolic resilience against ischemia, rather than direct inhibition of tissue excitability and depolarization, as the mechanism of reduced infarct volumes by meclizine.

### Synthesis and Characterization of (*S*)-Meclizine

**Figure 4.**
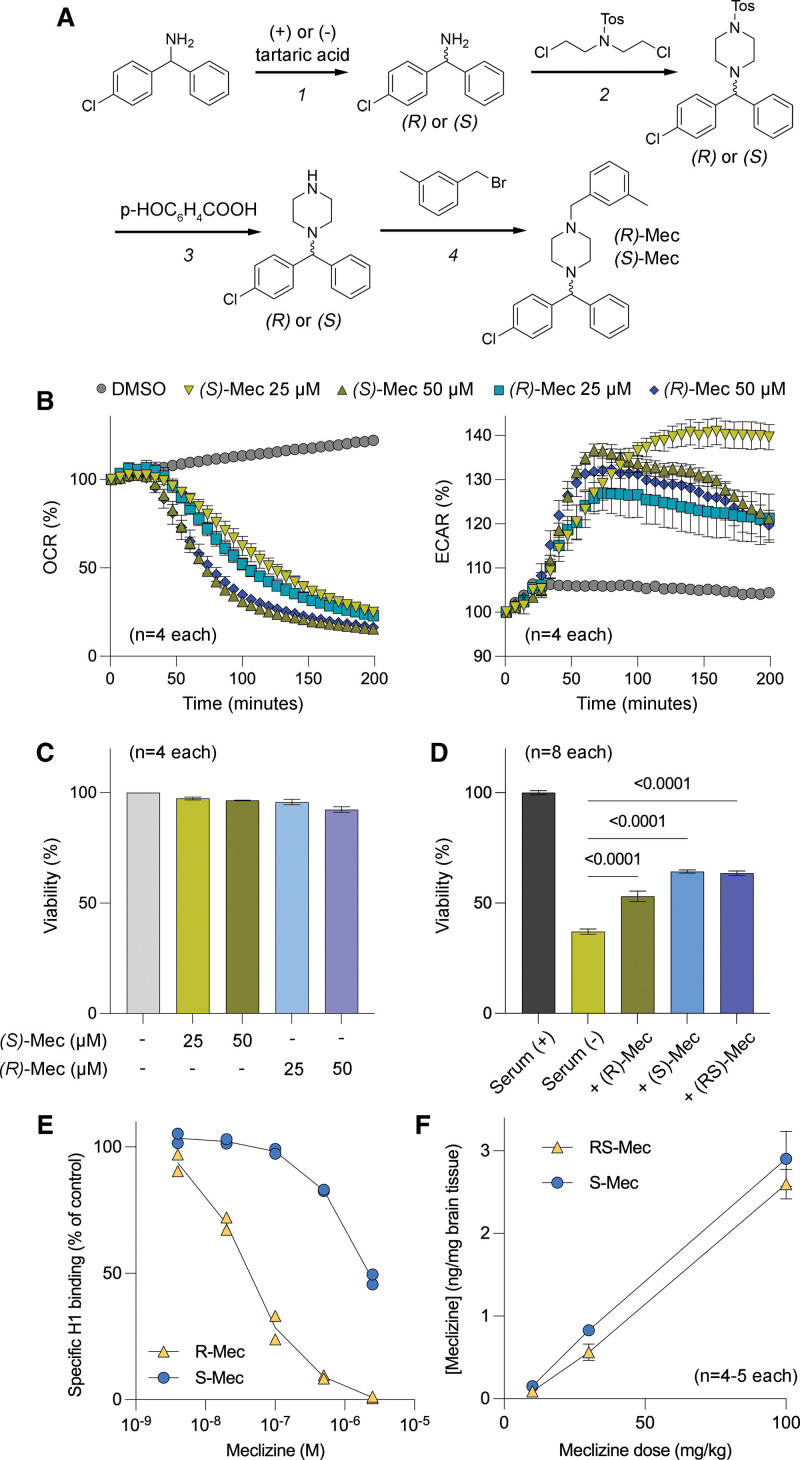
**Synthesis and functional characterization of (R)- and *(S*)-meclizine. A**, Chiral synthesis of *R* and *S* enantiomers of meclizine. **B**, Time course of oxygen consumption rate (OCR) and extracellular acidification rate (ECAR) of HEK-293MSR cells following 25 μM and 50 μM treatment with *(R*) and *(S*) enantiomers of meclizine. **C**, Viability of HEK-293MSR cells measured after 6 h of treatment with the indicated concentrations of (R)- and (*S*)- enantiomers of meclizine. **D**, Cell viability measured by ATP levels of striatal neurons cultured in the presence or the absence of fetal bovine serum and co-treatment with 50 μM of (R), (*S*), or racemate meclizine (n=8). **E**, The binding affinity of (R)- and (*S*)-meclizine for H_1_ histamine receptor. **F**, Mass spectrometry-based detection of meclizine and (*S*)-meclizine in the brain tissue of mice after 5-d treatment with the indicated concentrations of these drugs injected once daily.

Meclizine harbors a single chiral center and commercially available formulations are racemates, that is, contain an equal amount of *(R*)- and *(S*)-meclizine. The racemate is known to have several side effects that are attributed to its antimuscarinic and antihistaminic effects. Initial attempts to dose mice chronically with the racemate resulted in a loss of body weight (unpublished data). We performed the synthesis of optically active *(R*)- and *(S*)-meclizine to identify an enantiomer with reduced side effects (Figure 4A and Methods). We first compared the efficacy of *(R*)- and *(S*)-meclizine to attenuate cellular oxygen consumption rate in vitro in HEK293MSR cells. Maximal inhibition was observed after about 3 hours posttreatment (Figure 4B). Both enantiomers inhibited the oxygen consumption rate by ≈60% and 80% at 25 and 50 μM concentrations, respectively. Simultaneous measurement of the extracellular acidification rate in these cells showed a corresponding increase in the extracellular acidification rate, suggesting that these drugs shift cellular metabolism from oxidative to glycolytic (Figure 4B). Importantly, the viability of cells was not altered following a 6-hour treatment with drugs (Figure 4C). To demonstrate the cytoprotective effect of meclizine and its enantiomers in a more physiologically relevant cell type, we utilized striatal neuronal cell line derived from mutant huntingtin-expressing mice. These cells undergo rapid apoptosis upon serum withdrawal. We found that meclizine and its enantiomers partially but significantly improved the viability of striatal cells upon serum withdrawal (Figure 4D). In terms of antihistaminic effects, we found that *(S*)-meclizine exhibited a much weaker affinity for the H_1_ receptor than *(R*)-meclizine (Figure 4E), suggesting that the *S* enantiomer might not cause drowsiness typically associated with antihistaminic drugs. Lastly, we measured brain penetration of the racemate and *(S*)-meclizine after 5 once-daily intraperitoneal injections at 10, 30, and 100 mg/kg dose levels. We found that both *RS*- and *(S*)-meclizine reached equivalent brain concentrations at all dose levels (Figure 4F). Moreover, in pilot studies, mice could tolerate daily IP injections of 100 mg/kg/day of *(S*)-meclizine without overt impact on body weight (data not shown). These data showed that the (*S*) enantiomer possessed an improved toxicity profile compared with racemic meclizine.

### (*S*)-Meclizine Delays Anoxic Depolarization and Improves Outcomes After Transient Focal Cerebral Ischemia

**Figure 5.**
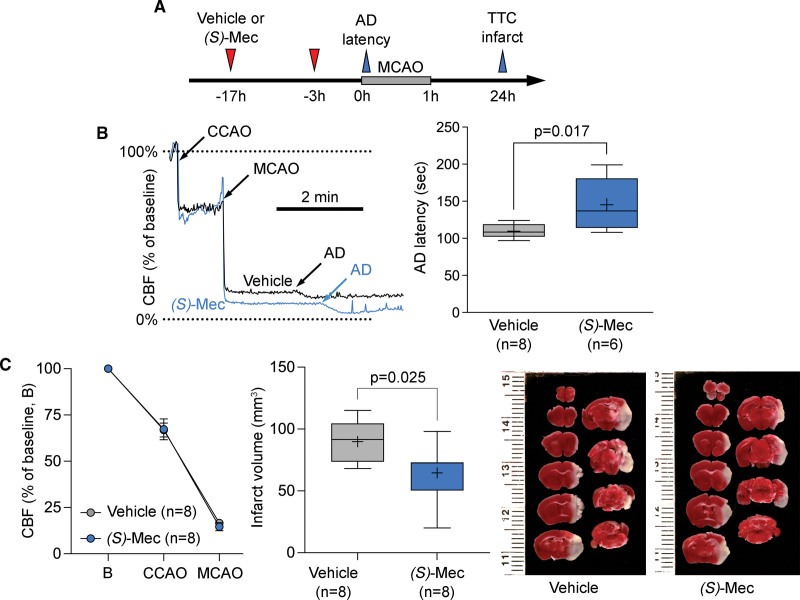
**Effects of *(S*)-meclizine on anoxic depolarization latency, cerebral blood flow, and infarct volume in the filament middle cerebral artery occlusion (MCAO) model. A**, Experimental timeline shows the treatment protocol and the timing of data collection for anoxic depolarization (AD) latency and infarct volume using triphenyl tetrazolium chloride (TTC) staining. **B**, A representative cerebral blood flow (CBF) tracing shows the common carotid artery occlusion (CCAO) and MCAO time, and the stepwise CBF reduction at the onset of AD in a vehicle and an (*S*)-meclizine animal. The right shows the prolonged AD latency in the (*S*)-meclizine arm (unpaired *t* test). **C**, The left shows nearly identical CBF reductions upon CCAO and MCAO between (*S*)-meclizine and vehicle arms. The right shows smaller infarcts in the (*S*)-meclizine arm (unpaired *t* test).

Having identified *(S*)-meclizine as a more selective inhibitor of mitochondrial respiration than the racemate, we next sought to evaluate its neuroprotective efficacy in ischemic stroke. Using the same therapeutic paradigm as with meclizine, we found that *(S*)-meclizine delayed AD onset (*P*=0.017), and reduced infarct volume (*P*=0.025) compared with the vehicle, without altering the CBF deficit (Figure 5). Neurological deficit scores were milder in the *(S*)-meclizine arm compared with the vehicle (1.00 [1.00 to 1.00] versus 2.00 [1.25 to 2.00], respectively; *P*=0.0406, Mann-Whitney *U* test). These data were consistent with equipotent inhibition of oxygen consumption rate by *R*- and *(S*)-meclizine and suggested that shifting the metabolism from mitochondrial respiration towards anaerobic glycolysis is a viable neuroprotective strategy in acute ischemic stroke.^6^

## DISCUSSION

Meclizine has emerged as a promising candidate therapeutic agent for prophylaxis against ischemia-reperfusion injury to the brain, heart, and kidney.^4,6^ It has also emerged as a promising neuroprotective agent for Parkinson disease,^16^ Huntington disease,^17^ and cisplatin toxicity in dorsal root ganglion neurons.^18^ The cytoprotective effects of meclizine have been attributed to its ability to attenuate mitochondrial respiration while shifting cellular metabolism towards glycolysis. However, as a first-generation antihistaminergic agent, it is expected to have unwanted side effects associated with its long-lasting activity that presents an obstacle in repurposing this drug for ischemic injury. To this end, here we report the synthesis and characterization of *(S*)-meclizine, which attenuates mitochondrial respiration with similar efficacy as the racemate but shows greatly reduced activity against the histamine receptor, minimizing potential side effects associated with the racemate, such as drowsiness. Mice tolerate high doses of (*S*)-meclizine. Our data also show that *(S*)-meclizine reduces infarct volume and improves outcomes in filament MCAO, the most commonly used translational model of transient focal ischemia worldwide that was also recently adopted by the National Institutes of Health Stroke Preclinical Assessment Network (SPAN).^19^

We provide the first direct evidence using ^18^F-FDG PET, in vivo, showing enhanced brain glucose uptake after treatment with meclizine, consistent with a shift in energy metabolism towards glycolysis. This reduced reliance on mitochondrial respiration has been the presumed mechanism of neuroprotection rendering cells more resistant to hypoxic and ischemic insults.^1–3^ The effect was most pronounced in the ischemic penumbra where it would have the greatest impact on tissue survival and persisted when uptake was corrected for the reduced CBF (ie, tracer delivery) by calculating the net tracer influx rate (K_i_). Glucose uptake in the contralateral brain also increased, albeit statistically nonsignificantly. Reduced uptake in the core and elevated uptake in the penumbra in the vehicle arm were consistent with previous reports using ^18^F-FDG PET in both experimental and human focal cerebral ischemia,^20–25^ increasing confidence in our technique.

A unique property of the brain is the sudden onset loss of neuronal membrane potentials when tissue ATP levels drop below a critical threshold during a hypoxic or ischemic insult.^26^ Also known as AD, this sudden but persistent drop in membrane resistance is considered an early marker for the transition to irreversible injury in the presence of sustained hypoxia or ischemia. The time it takes for the tissue to develop AD reflects the rate at which tissue ATP levels decline upon ischemia. Therefore, a delay in AD onset reflects the metabolic resilience of the tissue experiencing a hypoxic or ischemic insult. We have previously used this biomarker as a reflection of tissue resistance to ischemic injury^27^; however, AD latency is also affected by the overall excitability of the tissue (ie, hyperexcitable tissue is more susceptible to depolarizations) and meclizine could alter tissue excitability and thereby the AD latency. We ruled out this possibility by directly measuring the effect of meclizine on SD susceptibility and did not find any effect in this important control experiment. Altogether, these data strongly suggested that delayed AD latency by meclizine indeed reflected metabolic resilience.

Several preclinical studies have now demonstrated that metabolic shifting with meclizine can protect tissues from diverse forms of injury.^4,6,16–18,28^ In all of these studies, doses of meclizine higher than what is currently FDA approved have been required. At these higher doses, we would predict unwanted side effects from meclizine. In this study, we report the synthesis of (*S*)-meclizine as a single enantiomer that is as potent as the racemate in shifting metabolism from oxidative phosphorylation to glycolysis, with far less anti-histamine activity and better tolerated.

The ability of meclizine and (*S*)-meclizine to confer protection against ischemia-reperfusion injury likely involves both its ability to blunt respiration as well as its ability to boost ATP via glycolysis. A growing body of data suggests that during ischemia-reperfusion injury, the electron transport chain may undergo conformational changes and that with reperfusion, reactive oxygen species may be generated both by the forward flow of electrons as well as by reverse electron transport. As an inhibitor of respiration, meclizine would be predicted to blunt both forms of ROS from the electron transport chain, and its shift to glycolysis would provide bioenergetic benefit. We previously reported that meclizine could be blocking respiration by its ability to directly target the PCYT2 enzyme, which leads to an elevation in phosphoethanolamine levels,^5^ which we and others have shown can directly inhibit respiration in purified mitochondria. A subsequent article has shown that phosphoethanolamine may be directly blocking complex II of the respiratory chain.^29^ Consistently, direct administration of phosphoethanolamine was shown to recapitulate meclizine-induced protection both in vitro and in vivo in the kidney ischemia-reperfusion injury model.^6^ Prior work in cellular models of neurotoxicity has focused on the shift from respiration to glycolysis as being pivotal. In the current work, we have shown for the first time in vivo that meclizine is able to preserve ATP during ischemic injury by boosting glycolysis.

Clinically, the rapid metabolic preconditioning and the emergence of ischemic tolerance with only 2 doses of *(S*)-meclizine within less than a day opens a path for short-term preemptive prophylaxis in circumstances with temporally predictable elevated stroke risk (eg, surgical or endovascular procedures, delayed cerebral ischemia after aneurysmal subarachnoid hemorrhage). Indeed, such large target patient populations in need of short-term prophylaxis would make testing *(S*)-meclizine in clinical trials highly practical. If proven safe for chronic use, even longer-term prophylaxis with *(S*)-meclizine might be feasible in conditions such as progressive cerebrovascular insufficiency. Meclizine has also shown efficacy in experimental models of myocardial and renal ischemia^4,6^; therefore, the same principles could be extrapolated to other organ systems as well.

Of course, further translational steps must be taken to advance *(S*)-meclizine towards clinical testing, such as optimizing the therapeutic paradigm (eg, optimal dose, dose interval, duration of treatment), confirming persistent efficacy at later time points of assessment, and in comorbid animal models (eg, aging or hypertensive animals). However, given the known safety profile of the over-the-counter racemic formulation of meclizine, rapid translation of this new therapeutic paradigm may be possible.

## ARTICLE INFORMATION

### Acknowledgments

The authors thank Professor Eng H. Lo for his contributions during the preparation of this article.

### Sources of Funding

This work was supported by funding from the National Institutes of Health Award 5U01NS113443-03 to Dr Ayata and R01GM143630 to Dr Gohil.

### Disclosures

Drs Gohil, Ji, and Mootha are listed as coinventors on a patent issued to Massachusetts General Hospital on the therapeutic uses of (*S*)-meclizine. Dr Heidari reports compensation from Telix Pharmaceuticals for consultant services, all outside the submitted work. Dr Mootha reports compensation from 5am Ventures for consultant services, all outside the submitted work. Dr Ayata reports grants from Praxis, compensation from Neurelis, Inc, for other services, and grants from Takeda Pharmaceutical Company, all outside the submitted work. The other authors report no conflicts.

### Supplemental Material

ARRIVE Checklist
